# A three month controlled intervention of intermittent whole body vibration designed to improve functional ability and attenuate bone loss in patients with rheumatoid arthritis

**DOI:** 10.1186/1471-2474-15-403

**Published:** 2014-11-29

**Authors:** Alessandra Prioreschi, Mohammed Tikly, Joanne A McVeigh

**Affiliations:** Exercise Physiology Laboratory, School of Physiology, Faculty of Health Sciences, University of the Witwatersrand, Johannesburg, South Africa; Division of Rheumatology, Department of Medicine, Chris Hani Baragwanath Academic Hospital, University of the Witwatersrand, Johannesburg, South Africa

**Keywords:** Rheumatoid arthritis, Whole body vibration, Functional ability, Bone mineral density, Physical activity levels

## Abstract

**Background:**

Rheumatoid arthritis (RA) is a chronic autoimmune condition that results in pain and disability. Patients with RA have a decreased functional ability and are forced into a sedentary lifestyle and as such, these patients often become predisposed to poor bone health. Patients with RA may also experience a decreased health related quality of life (HRQoL) due to their disease. Whole body vibration (WBV) is a form of exercise that stimulates bone loading through forced oscillation. WBV has also been shown to decrease pain and fatigue in other rheumatic diseases, as well as to increase muscle strength. This paper reports on the development of a semi randomised controlled clinical trial to assess the impact of a WBV intervention aiming to improve functional ability, attenuate bone loss, and improve habitual physical activity levels in patients with RA.

**Methods/Design:**

This study is a semi randomised, controlled trial consisting of a cohort of patients with established RA assigned to either a WBV group or a CON (control) group. Patients in the WBV group will undergo three months of twice weekly intermittent WBV sessions, while the CON group will receive standard care and continue with normal daily activities. All patients will be assessed at baseline, following the three month intervention, and six months post intervention. Main outcomes will be an improvement in functional ability as assessed by the HAQ. Secondary outcomes are attenuation of loss of bone mineral density (BMD) at the hip and changes in RA disease activity, HRQoL, habitual physical activity levels and body composition.

**Discussion:**

This study will provide important information regarding the effects of WBV on functional ability and BMD in patients with RA, as well as novel data regarding the potential changes in objective habitual physical activity patterns that may occur following the intervention. The sustainability of the intervention will also be assessed.

**Trial registration:**

PACTR201405000823418 (19/05/2014).

**Electronic supplementary material:**

The online version of this article (doi:10.1186/1471-2474-15-403) contains supplementary material, which is available to authorized users.

## Background

Rheumatoid Arthritis (RA) is the most common, chronic autoimmune disease. It results in joint swelling, tenderness and destruction of the synovial joints, pain, severe disability, and decreased functional ability [1]. RA occurs in 1% of people worldwide, with the prevalence being two times higher in women than in men [2].

The health assessment questionnaire (HAQ)- a disease specific measure of functional ability in RA, is a widely used outcome measures for RA [3]. A large qualitative study conducted in female patients with RA found that patients report pain and decreased functional ability as having the most widespread effect on their daily lives [4]. Inability to perform normal daily activities not only decreases health related quality of life (HRQoL), but also further perpetuates a sedentary lifestyle. HAQ scores have been shown to be improved in patients with RA who have participated in exercise interventions aimed at increasing their regular levels of physical activity [5].

Physical activity as a functional measure of HRQoL can be difficult to assess. Commonly used questionnaires and recall diaries are subjective [6] and rely on fluency in the English language. Accelerometers are thus growing in popularity as an objective way to measure physical activity, especially in healthy populations [7]. Accelerometers are small, unobtrusive and comfortable, and measure acceleration of the limb to which they are attached by detecting low frequency (0.5-3.2 Hz) gravitational forces (0.05-2.0 g) [7]. Acceleration is directly proportional to muscle forces generated, which is proportional to energy expenditure [8]. This theory along with an inbuilt algorithm allows for the conversion of acceleration into activity counts. These counts can be classified into thresholds, indicating light, moderate or heavy intensity levels [8]. Accelerometers are able to track intensity, duration and frequency of an activity without relying on patient recall [9]. Actical accelerometers in particular, can detect varying levels of activity, being able to detect lower level activities and movement in multiple planes [7] and may therefore be an ideal tool for measuring physical activity and sedentary behaviour in patients with RA, where most movement is functional and of a low frequency and intensity, and therefore unlikely to be reported accurately using self report measures. Indeed accelerometry has already been used to this effect in other rheumatic diseases [10]. Although there is a paucity of research which has objectively assessed physical activity levels in people with RA, our group, as well as Semanik et al. [11] have recently demonstrated that sedentary behaviour as assessed using accelerometery is indeed more prevalent in patients with RA when compared to their healthy counterparts [12].

Patients with RA have lower bone mineral density (BMD) at the hip, spine, and whole body than age matched controls [13]. Lower BMD and higher incidence of osteoporosis in patients with RA may be as a result of the presence of circulating inflammatory cytokines inherent to the disease, the decreased mobility of these patients, or due to certain medications taken to treat the disease such as corticosteroids. Osteoporosis in RA can be generalised or peri-articular in nature. Studies have shown that the majority of bone density loss in RA occurs in the first six months of disease [14]. Furthermore, patients with higher RA disease activity have been shown to exhibit a greater loss in BMD, as well as higher indices of bone metabolism compared to those with lower disease activity. Mobility and functional ability have been correlated with BMD; as have age, stature, and sex independently of RA disease [15, 16].

The skeleton transforms it’s mass and morphology according to individual activity levels and forces placed upon the bone [17]. Furthermore a minimum effective strain must be placed upon bone in order for remodeling to occur [18]. A sedentary individual does not place sufficient strain on the skeleton, and bones are thus remodeled in a direction that promotes bone loss. Patients with RA are therefore at increased risk of osteoporosis due to inflammatory processes inherent to their disease; as well as their sedentary lifestyle [19]. Bone mass in RA can be modified using treatments designed to increase bone mass; decrease RA disease activity; and by increasing physical activity or decreasing sedentary behavior sufficiently in order to increase bone loading and remodeling. Research has shown that exercises aimed at increasing or attenuating loss of BMD should be dynamic- comprised of short and vigorous bouts of high impact exercise incorporating rest periods [20], yet this type of exercise is usually not feasible in patients with a chronic, disabling pain condition such as RA.

Exercise interventions making use of light aerobic activity, strength training, and stretching, have produced varied results in cohorts of RA patients. Most studies show exercise improves physical fitness and muscle strength with either no change or improved disease activity outcomes [21], however these interventions have not specifically addressed the problem of low bone mass in these patients. Whole body vibration (WBV) is a potential novel exercise intervention for people with RA. WBV therapy is an exercise whereby a mechanical vibration platform produces energy via forced oscillation. The vibratory waves are then transferred to an individual via propagation through the feet, legs, trunk and finally, the head [22]. Although the exact mechanisms whereby WBV therapy increases BMD are unclear, it is likely that there are multiple mechanisms at play. WBV has been shown to activate fluid flow in the caniliculi and lacunae of bone matrix in rats [23], in a manner proportional to loading frequency. This fluid flow creates shear stress on the plasma membrane of osteocytes, bone lining cells, and osteoblasts, which therefore respond accordingly [20]. WBV thus activates mechanotransduction in bone and stimulates osteogenesis [23]. Furthermore, muscle forces have been shown to exert the greatest osteogenic stimulus on bone, and the generation of these forces through vibration stimulus is likely a contributor to the skeletal adaptations that occur [24]. Vibratory stimuli must sufficiently load bones in order to increase bone deposition.

WBV has been used to treat osteoporosis in otherwise healthy populations with low BMD [23], older individuals [25], postmenopausal women [26], athletes at risk of osteoporosis [27], as well as in other diseased populations [28] with mainly positive results including increased or attenuated loss of BMD, increases in muscle strength, improved proprioception and balance, and decreased pain and fatigue levels. Furthermore, WBV therapy has been shown to increase peripheral blood flow [29] as well as cardiovascular performance [30], and could therefore have an effect on cardiovascular health. Trans et al. [31], used WBV in patients with knee osteoarthritis and found 8 weeks of twice weekly vibration training to significantly improve knee strength in these patients, but did not assess BMD. Alentorn-Geli et al. [32] used a dynamic and static WBV protocol, twice weekly for 6 weeks in a group of female patients with fibromyalgia. Patients in this study were divided into a control group who underwent no therapy, an exercise group who underwent standard RA exercise therapy only, and a WBV group who underwent WBV training on top of the standard exercise therapy. The authors of this study found that the WBV protocol significantly improved fatigue scores, as well as pain scores in comparison to the exercise group and the control group, however no measures of BMD were taken. Other studies have shown WBV therapy to have no effect on BMD in healthy adults [33], or on fatigue or pain levels, balance, or strength [34].

WBV has not, to our knowledge, been used as an exercise intervention for RA, yet it may be a feasible means to increase functional ability in these patients. Since patients with RA are already at risk for developing osteoporosis, and are therefore at greater risk of fracture; WBV could also potentially be a means to attenuate the progressive loss of BMD observed in patients with RA, without the need for a vigorous exercise programme. Previous studies that have used WBV in other chronic inflammatory or pain conditions do not suggest that WBV would elicit any adverse effects in this population. With this background in mind, the aims of this study are primarily to determine the effects of a WBV programme on functional ability in patients with established RA in comparison to a control group of patients, as well as to determine any effects WBV therapy may have on BMD, disease activity, physical activity levels, HRQoL, or body composition in these patients.

## Methods

### Study design

**Figure 1:**
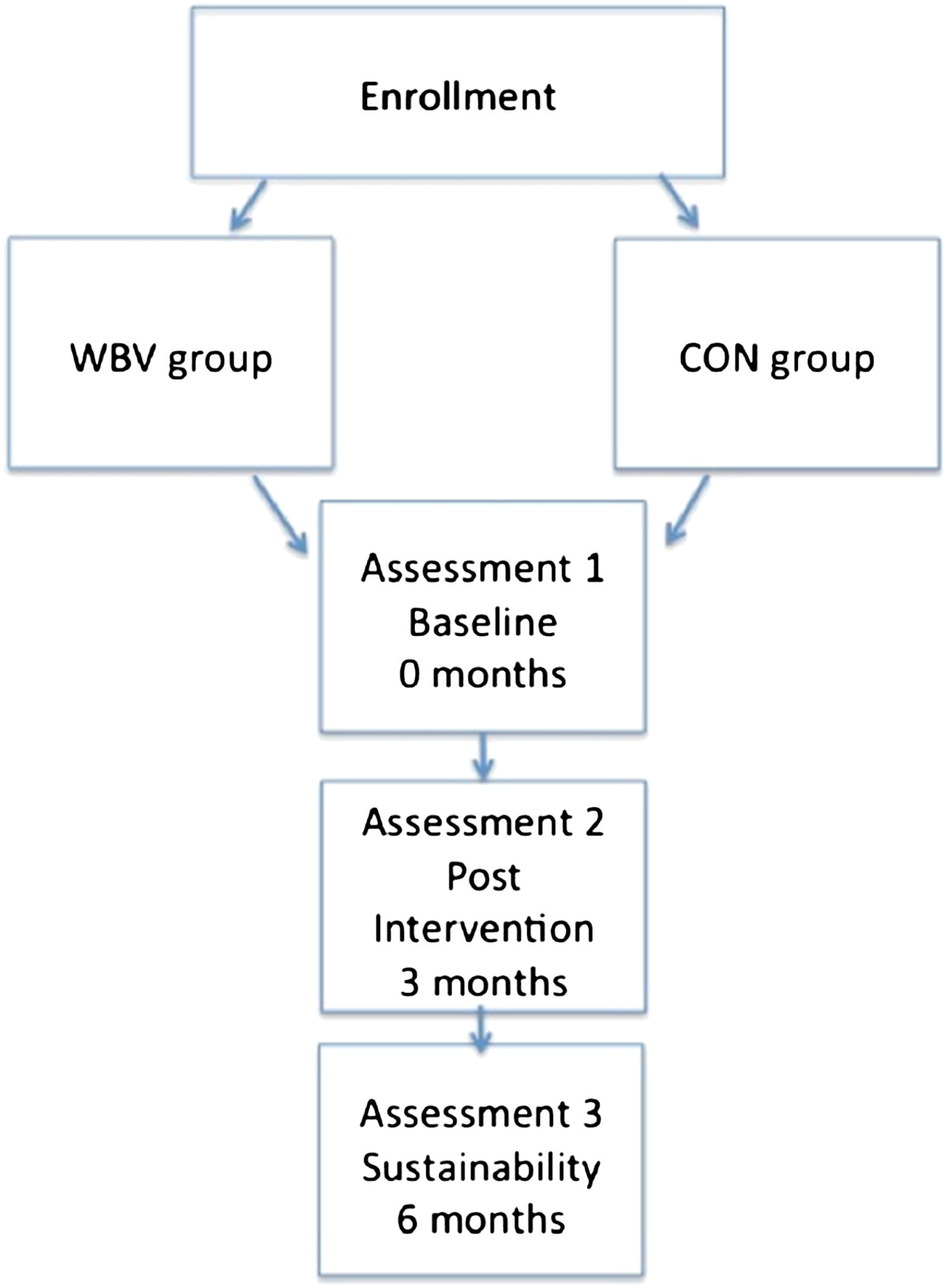
**Flow diagram of study design.**

A semi randomised, single blinded, controlled, two-group parallel design in accordance with the 2010 CONSORT guidelines will be used; with patients being allocated to either group in an alternating manner upon enrollment. Patients in the WBV group will begin a WBV therapy intervention programme for a three month period, while those in the CON group will continue to receive standard care for the three month period. All patients will be assessed at three time points, as shown in Figure 1.

### Participants

Fifty participants will be recruited from the Rheumatology Clinic at the Chris Hani Baragwanath Academic Hospital in Soweto, South Africa. Recruitment will include the outpatients attending the clinic at the quarterly check-up via a brief interview following dissemination of study information. Consenting patients will be included if they are older than 18 years, have been diagnosed with RA (according to the 1987 ACR criteria [35]) at least three years previously, are on stable drug therapy (prednisone <10 mg/day), and had been for at least three months previously. Patients will be excluded if they are HIV+, are using bisphosphonates or corticosteroids, have any co-morbidities that could potentially impact on physical activity levels, are using assistive walking devices, have previously had hip or knee joint replacement surgery, and if they are pregnant.

### Ethical consideration

This study complies with the international ethical guidelines for a clinical study. Ethical approval has been obtained from the Human Research Ethics Committee of the University of the Witwatersrand (M130113). Patients will be required to read and sign informed consent, and revocation of consent will not detriment the patients in any way.

### Trial registration

This study has been registered with the Pan African Clinical Trial Registry and has the trial number PACTR201405000823418 (http://www.pactr.org).

### Intervention and control

All vibration training will be performed on standard power plates (DKN XG 5.0, DKN Technology, California, USA) under the supervision of the primary investigator. Vibration training will consist of 24 total sessions (performed twice weekly for 12 weeks); in intermittent bouts of 60 seconds on the plate and 30 seconds off the plate, repeated 10 times (this protocol was designed to stimulate greater osteogenic responses due to the constant stimulus to the mechanoreceptors [27]). Patients will be required to stand on the plates, barefoot and with knees slightly bent, holding firmly to the bars. Vibration plates will be set at a constant frequency of 30Hz and amplitude of 3 mm in order to maximize the osteogenic and muscle activation effects of the therapy [27]. The CON group will continue to receive standard care for the intervention period, and will be instructed to continue with their normal daily activities for the three month period.

### Sample size

A sample size calculation (ß = 0.10) based on detecting a minimum clinically important difference of 0.22 in HAQ score (using an SD of 0.19) [36] between the two groups, showed that at a 5% level we would require a sample of 8 participants in each group with a power of 90%.

### Outcome measures

All outcomes will be measured at baseline, and reassessed after the three month intervention, as well as again three months post intervention. The primary outcome of this study will be an improvement in functional ability as assessed by the HAQ. The secondary outcomes will be attenuation in loss of BMD at the hip, as well as improvements in subjective pain scores, SF-36, CDAI, and objective habitual physical activity levels (Table 1).

**Table 1:** **Summary of outcome measures and respective methodology**

Outcome		Measurement method	Time point (months)
**Primary**	Functional ability	Health assessment questionnaire	0,3,6
**Secondary**	BMD	DXA	0,3,6
	Habitual physical activity	Accelerometry (Actical worn on the hip)	0,3,6
	Disease activity	CDAI assessment	0,3,6
	Pain	Self reported via Lickert scales	0,3,6 as well as at each session of WBV therapy
	Fatigue	Self reported via Lickert scales	0,3,6
	Body composition and anthropometry	DXA, standard scale, and stadiometer	0,3,6
	Acute inflammation	CRP	0,3,6

### Primary

#### Functional ability

Patients will be asked to complete the modified Health Assessment Questionnaire (HAQ) [37], which is a RA specific questionnaire that assesses functional ability by providing a score of functionality between 0 and 3, where 0 indicates good functionality and 3 indicates severe functional disability.

### Secondary

The secondary outcomes of the study are attenuation in loss of BMD, as well as any improvements in HRQoL, disease activity, habitual physical activity or body composition measurements. These will be assessed as follows:

#### BMD

All patients will be assessed, for site specific areal BMD at the left hip, lumbar spine (L1-L4), and whole body using Dual X-Ray Absorbtiometry (DXA). T and Z scores will then be calculated according to reference values. All scans will be performed by the same qualified technician on the same machine (Hologic QDR 4500A, Hologic, Boston, USA). The machine is routinely calibrated, and a phantom spine will be scanned daily to determine coefficients of variation of the machine. The technician will be blinded as to the grouping of participants during the study. According to previous studies examining bone loss over time in age matched female patients with RA [38], and the effects of exercise interventions on this type of bone loss [39]; we expect a decrease of approximately 0.33% in hip BMD in the control group, and for this loss to at least be attenuated in the WBV group.

#### Physical activity

At the first assessment, during the last week of the intervention, and again at the follow up assessment, patients will be fitted with an Actical (Respironics Inc., Murrysville, PA, USA) accelerometer (for the assessment of habitual physical activity) worn on a Velcro belt on the hip for a one week period. Patients will be instructed to wear the accelerometer all day, and to remove the device only while sleeping, bathing or showering. Patients will then return the accelerometer to the clinic one week later. Actical data are reported in one minute epochs and raw data are reduced by removing full days of non-wear time by visual inspection of the data where a full day of consecutive zero activity counts are observed. Sleep time is also removed by visual inspection of the data, and the remaining data will be considered as wear time. Thereafter, a valid day will require at least ten hours of wear time per day, and at least four valid days will be required for inclusion in the final analysis [40]. Data will be reported as average activity counts per day, as well as percentage of time spent in sedentary, light, moderate and vigorous activity thresholds, as calculated by the inbuilt algorithm on the Actical software. The number of bouts of activity, as well as the number of breaks in sedentary activity per day will also be reported, the methodology of which has been explained previously [41]. Participants will be fitted with the same Actical at each visit in order to minimise inter-device variability. This will allow for the assessment of habitual physical activity patterns before and after the intervention.

#### Disease activity

Patients will be assessed for disease activity using the Compound Disease Activity Index (CDAI) [42] which provides a score comprised of tender joint count (TJC), swollen joint count (SJC), patient global assessment (PGA), and physician global assessment (MGA) calculated as follows:

CDAI=TJC+SJC+PGA+MGA

This score allows for the classification of patients according to the severity of their disease where a score <2.8 indicates remission, a score <10 indicates moderate disease activity, a score <22 indicates moderate disease activity, and a score >22 indicates severe disease activity. The physician will be blinded as to the grouping of participants during assessments.

#### Inflammation

Patients will be assessed for acute inflammation by measuring C-reactive protein (CRP) concentration from a 10 ml venous sample.

#### Pain

At each assessment, patients will be asked to rate their pain levels over the previous week using a Lickert scale ranging from 0–5, where a score of 0 indicates no pain and a score of 5 indicates unbearable pain. Furthermore, at each WBV session, patients will be asked to rate their pain levels on that day using the same Lickert scale.

#### Fatigue

At each assessment, patients will be asked to rate their fatigue levels over the previous week using a Lickert scale ranging from 0–5, where a score of 0 indicates not feeling tired at all, and a score of 5 indicates the most tired ever felt.

#### Anthropometry

Height and weight will be measured to the nearest cm and kg respectively using a standard stadiometer and scale, with patients barefoot and wearing minimal clothing. Thereafter body mass index (BMI) will be calculated.

#### Body composition

Body composition, including fat mass and lean muscle mass will be taken from the DXA scan. Percentage body fat and percentage lean muscle mass will then be calculated. Appendicular lean mass (ALM) will also be calculated and used to classify those patients with sarcopenia.

### Statistical analysis

Statistical analysis will be carried out using Stata version 12/IC 12.0. All data will be presented as mean ± SD, and a p value ≤0.05 will be considered significant. Data will be analysed on an intention to treat basis. Student’s unpaired t-tests will be used to compare absolute, as well as change in HAQ, BMD, physical activity, patient characteristics, and RA disease activity data between the WBV group and CON group at baseline. Cohen’s D effect sizes will be calculated to determine the magnitude of effects observed.

### Potential obstacles

Potential obstacles include adherence to the protocol, and attempting to minimise the attrition rate of the study. Participants will be in regular contact with the primary investigator who will attempt to minimise attrition, as well as follow up on patient’s health on a weekly basis. The short duration of the study, as well as the intermittent design of the study, lead us to expect an attrition rate of approximately 20% according to a meta-analysis by Linke et al. in 2009 [43]. There is the potential that WBV may cause adverse effects or may not be tolerated in some participants; however the primary investigator will observe every WBV session, and assess pain levels at each WBV session in order to monitor this. It is also possible that WBV will have no effect on the outcomes measured.

## Discussion

The present study will contribute to the current field of rheumatology by potentially providing a non-pharmacological means to improve functional ability and attenuate the loss of BMD associated with RA. This study may provide a safe, sustainable exercise intervention for these patients that could potentially improve certain aspects of disease activity, as well as HRQoL and habitual physical activity.

The advantages of the present study over previous exercise interventions in RA include, firstly, the use of a novel therapy in RA. WBV therapy has not previously been used in patients with RA (to the best of our knowledge), and could provide a safe and easy form of exercise for patients who are often unable to participate in strenuous activities. Furthermore, the application of an intermittent WBV programme could potentially exhibit the added benefit over the previous HRQoL and strength benefits seen following WBV therapy in other rheumatic diseases, of attenuating BMD loss in these patients. Very few studies have focused exercise interventions on improving or attenuating the loss in BMD in this population, despite the very high prevalence of osteoporosis that exists. Usually, interventions designed to increase BMD are dynamic and strenuous, which is not feasible in an RA population. WBV therapy could provide a solution to this problem.

Secondly, the use of an objective measurement of physical activity could further elucidate the benefits of the WBV intervention by providing an accurate, and detailed description of changes that may occur during and following the WBV intervention. Accelerometry allows for the novel examination of changes in patterns of habitual physical activity in this population following the intervention, which will help elucidate which thresholds of physical activity are affected by WBV therapy. Previous research conducted by the authors has shown by using accelerometry for the first time in this population as a means to compare physical activity levels to healthy control participants, that patients with RA are extremely sedentary, and that patients with higher levels of physical activity fare better on certain disease activity and HRQoL outcomes [12]. The potential ability of WBV therapy to increase physical activity levels could therefore attribute to any changes seen in functional ability, BMD, disease activity and HRQoL in the present study.

Lastly, the inclusion of a post intervention assessment allows the sustainability of the present protocol to be examined. If any changes are observed in any of the primary or secondary outcomes of the present study, it is important to be able to report on whether these changes will be sustained after cessation of the intervention, thereby adding strength to the feasibility of the intervention.

## Authors’ original submitted files for images

Below are the links to the authors’ original submitted files for images. Authors’ original file for figure 1
