# Supplementary figures and images for: A three month controlled intervention of intermittent whole body vibration designed to improve functional ability and attenuate bone loss in patients with rheumatoid arthritis
Source: BMC Musculoskelet Disord. 2014 Nov 29;15:403. doi: 10.1186/1471-2474-15-403 (PMC4265489; doi:10.1186/1471-2474-15-403)

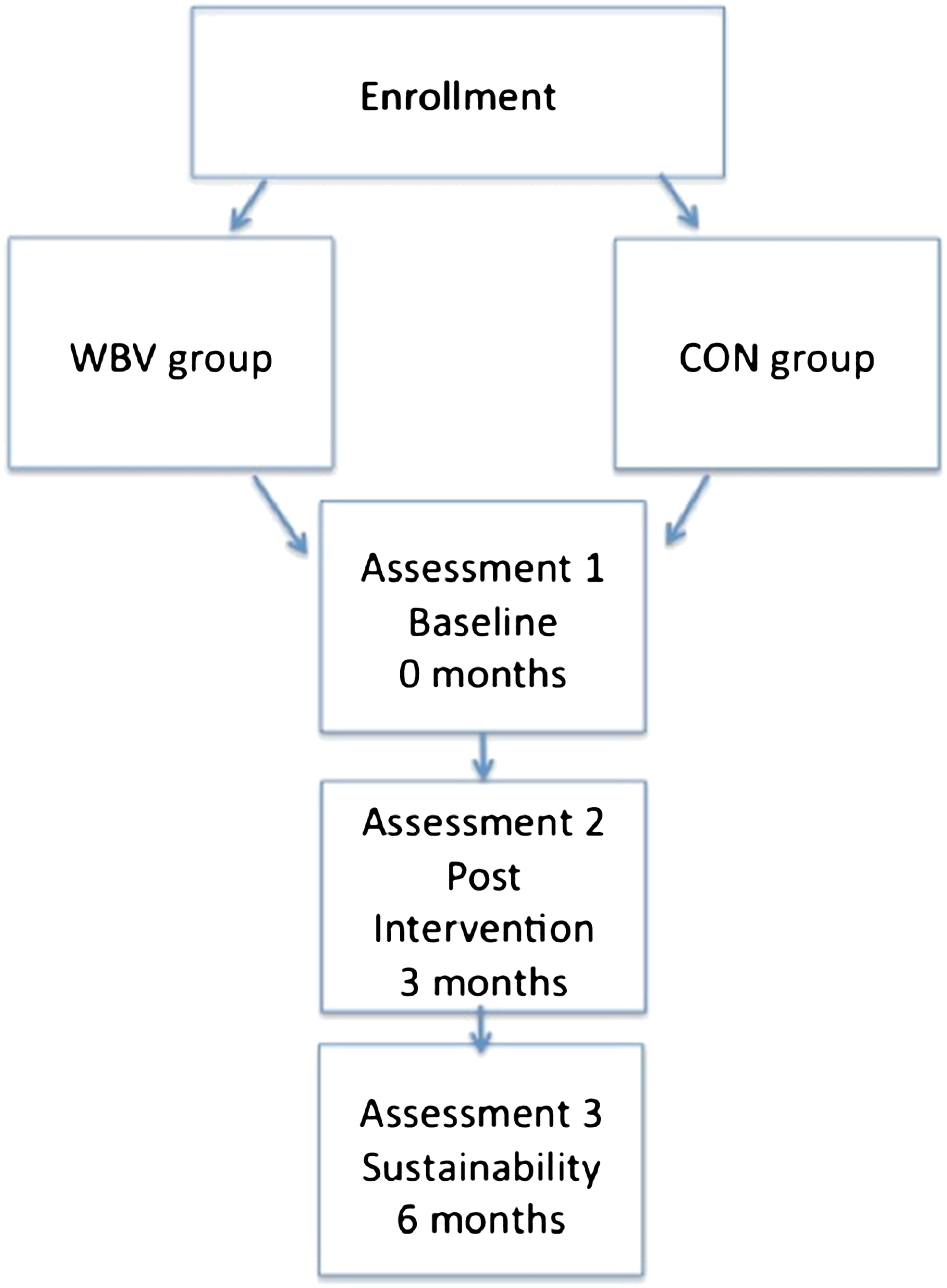

Supplement: Supplementary file 1 — Authors’ original file for figure 1 [file 12891_2014_2344_MOESM1_ESM.tif]
